# *Loranthus tanakae* Franch. and Sav. Attenuates Respiratory Inflammation Caused by Asian Sand Dust

**DOI:** 10.3390/antiox13040419

**Published:** 2024-03-29

**Authors:** Se-Jin Lee, So-Won Pak, A Yeong Lee, Woong-Il Kim, Sung-Wook Chae, Young-Kwon Cho, Je-Won Ko, Tae-Won Kim, Jong-Choon Kim, Byeong Cheol Moon, Yun-Soo Seo, In-Sik Shin

**Affiliations:** 1 BK21 FOUR Program, College of Veterinary Medicine, Chonnam National University, 77 Yongbong-ro, Buk-gu, Gwangju 61186, Jeollanam-do, Republic of Korea; 218729@jnu.ac.kr (S.-J.L.); dvmpsw@jnu.ac.kr (S.-W.P.); dvmwoong@jnu.ac.kr (W.-I.K.); toxkim@jnu.ac.kr (J.-C.K.); 2 Herbal Medicine Resources Research Center, Korea Institute of Oriental Medicine, 177 Geonjae-ro, Naju-si 58245, Jeollanam-do, Republic of Korea; lay7709@kiom.re.kr (A.Y.L.); bcmoon@kiom.re.kr (B.C.M.); 3 Department of Biotechnology, The Catholic University of Korea, 43 Jibong-ro, Wonmi-gu, Bucheon-si 14662, Gyeonggi-do, Republic of Korea; 4 Department of Biomedical-Chemical Engineering, The Catholic University of Korea, 43 Jibong-ro, Wonmi-gu, Bucheon-si 14662, Gyeonggi-do, Republic of Korea; 5 KM Convergence Research Division, Korea Institute of Oriental Medicine, 1672 Yuseong-daero, Yuseong-gu, Daejeon 34054, Chungcheongnam-do, Republic of Korea; sunguk.chae@kitox.re.kr; 6 Center for Companion Animal New Drug Development, Jeonbuk Branch, Korea Institute of Toxicology (KIT), 30 Baekhak1-gil, Jeongeup-si 53212, Jeollabuk-do, Republic of Korea; 7 College of Health Sciences, Cheongju University, 298 Daesung-ro, Sangdang-gu, Cheongju-si 28503, Chungbuk, Republic of Korea; petmen@cju.ac.kr; 8 BK21 FOUR Program, College of Veterinary Medicine, Chungnam National University, 99 Daehak-ro, Daejeon 34134, Chungcheongnam-do, Republic of Korea; rheoda@cnu.ac.kr (J.-W.K.); taewonkim@cnu.ac.kr (T.-W.K.)

**Keywords:** *Loranthus tanakae* Franch. and Sav., Asian sand dust, airway inflammation, network pharmacology, NF-κB, HO-1

## Abstract

Asian sand dust (ASD), generally produced in East Asia, including China, Japan, and Korea, directly leads to the development of pulmonary disease and exacerbates underlying pulmonary diseases. *Loranthus tanakae* Franch. and Sav. is a traditional herbal medicine applied to improve various inflammatory conditions. Here, we evaluated the curative properties of *L. tanakae* ethanol extract (LTE) against pulmonary inflammation caused by ASD. Additionally, to investigate the mechanism of action of LTE, we performed network pharmacological analysis. ASD was administrated on day 1, 3, and 5 by intranasal instillation, and LTE was orally administered for 6 days. Administration of LTE significantly decreased inflammatory cytokines and the number of inflammatory cells in bronchoalveolar lavage fluid, which was accompanied by a decrease in inflammatory cell accumulation in pulmonary tissue. Administration of LTE decreased the expression of cyclooxygenase2 and matrix metalloproteinase-9 in mice exposed to ASD with the decline in p65 phosphorylation. Additionally, administration of LTE significantly elevated hemeoxygenase (HO)-1 expression in the pulmonary tissue of mice exposed to ASD. These results were consistent with the data of network pharmacological analysis. This experiment showed that LTE attenuated pulmonary inflammation caused by ASD via inhibition of NF-κB and elevation of HO-1. Therefore, LTE may have potential as a therapeutic agent to treat pulmonary inflammation caused by ASD.

## 1. Introduction

Asian sand dust (ASD) is considered as an important social problem that threatens human health due to its harmful components [1]. Generally, ASD is called yellow dust or China dust and mainly occurs during spring and autumn in East Asia, including China, Japan, and Korea [2]. ASD originates from the deserts of Mongolia and China and affects East Asia due to the air current [3,4]. Furthermore, there are continuously elevated contents of particulate matters in ASD with increasing industrialization; thereby, ASD has led to disorders in various organs, such as the eyes, lung, and skin, and aggravated underlying respiratory diseases [5]. In particular, ASD induces airway inflammation via the release of inflammatory cytokines such as interleukin tumor necrosis factor (TNF)-α, (IL)-1β, and IL-6, resulting in the infiltration of inflammatory cells into ASD-exposed lesions [6]. Furthermore, ASD exposure in patients with asthma or chronic obstructive pulmonary disease exacerbates the degrees of underlying diseases throughout the activation of the inflammatory process [7]. However, although the riskiness induced by ASD exposure is continuously elevated, there has been hardly any development of therapeutics to control the respiratory disorder caused by ASD exposure.

*Loranthus tanakae* Franch. and Sav. has been applied to treat inflammatory diseases, particularly inflammatory respiratory diseases, as a conventional herbal remedy in China, Japan, and Korea [8]. According to previous studies, *L. tanakae* possesses various pharmacological activities, such as anti-inflammatory, antioxidant, and anticancer properties, which are associated with its ingredients, including afzelin, quercitrin, and rhamnetin 3-O-α-rhamnoside [9,10]. Furthermore, *L. tanakae* effectively suppresses airway inflammation caused by exposure to cigarette smoke. These documents indicate that *L. tanakae* may have therapeutic potential to treat inflammatory pulmonary disease. Considering this evidence, we predict that *L. tanakae* may effectively suppress airway inflammation induced by ASD exposure.

Thus, we detected the ingredients of *L. tanakae* ethanol extract (LTE) by means of high-performance liquid chromatography (HPLC) and explored the curative properties of LTE on an ASD-induced pulmonary inflammation model. To investigate the mechanism of action of LTE, we analyzed specific protein expression according to the data from network pharmacological analysis.

## 2. Materials and Methods

### 2.1. Plant Material

The aerial parts of *L. tanakae* were obtained from a herbalist in Jeongseon, Gangwondo in South Korea (voucher specimen No. 2-16-0335). The plant material was the same as in our previous study [10]. The results of analysis of four components, including afzelin, quercitrin, rhamnocitrin 3-rhamnoside, and rhamnoside 3-rhamnoside, in LTE were described in a previous study [10].

### 2.2. Small Molecules and Potential Target Genes

The chemical profiles of the main components of *L. tanakae* and related genes have been described in our previous report [10]. Potential target genes related to “pneumonia” were searched using the GeneCards: Human Gene Database (https://www.genecards.org/, version 5.14, accessed on 14 April 2023). The corresponding target gene information was searched in the UniProt database (https://www.uniprot.org/, accessed on 14 April 2023). Potential target genes were selected according to the intersection of genes associated with small molecules and disease-linked genes [10].

### 2.3. Protein–Protein Interaction (PPI)

Various proteins have numerous reactions in the human body because there are many protein–protein interactions in the human body [11,12]. PPI analyses were performed using the STRING database (https://string-db.org/, version 11.5, accessed on 14 April 2023) with a medium confidence score (≥0.400). The PPI’s topology was analyzed and visualized using Cytoscape version 3.7.2 (https://cytoscape.org/, accessed on 14 April 2023), with node colors of red (large nodes) and blue (small nodes) [13].

### 2.4. Signaling Pathway Analysis

Signaling pathway analyses were performing using the DAVID (The Database for An-notation, Visualization and Integrated Discovery; https://david.ncifcrf.gov/, accessed on 24 April 2023) and KEGG (Kyoto Encyclopedia of Genes and Genomes; https://www.genome.jp/kegg/, accessed on 25 April 2023).

### 2.5. Physicochemical Characterization of Asian Sand Dust

Asian sand dust (ASD) was obtained from Power Technology Inc. (Arden Hills, MN, USA) and consisted of 50% JIS Z8901 Class 8 (SiO_2_, Al_2_O_3_, Fe_2_O_3_, MgO, CaO, TiO_2_) and 50% natural SiO_2_. The primary size, morphology, and purity of ASD were evaluated as previously described [2].

### 2.6. Experimental Animals and ASD Induced Airway Inflammation Model Protocol

The animal model was established as previously described [2]. The animals (male C57BL/6 mice, 6 weeks old, Samtako, Osan, Republic of Korea) were randomly divided into 4 groups as follows: control group (CON, PBS intranasal exposure + PBS oral administration), ASD group (ASD, ASD intranasal exposure + PBS oral administration), and LTE groups (LTE 50 and 100, ASD intranasal exposure + LTE 50 and 100 mg/kg oral administration, respectively). ASD intranasal exposure (40 mg/kg) was conducted on days 1, 3, and 5 under slight anesthesia. LTE was administrated daily from days 1 to 6 by oral gavage before ASD exposure.

### 2.7. Analysis of Bronchoalveolar Lavage Fluid and Serum

The animals were sacrificed 48 h after the last ASD exposure, and tracheostomy was performed to collect bronchoalveolar lavage fluid (BALF) according to a previous study [10]. The supernatant of BALF was used to measure tumor necrosis factor (TNF)-α, interleukin (IL)-1β, and IL-6 using ELISA kits (BD Biosciences, San Jose, CA, USA). The pellets of BALF were diluted with PBS and then used to count the inflammatory cells of BALF. The number of inflammatory cells in BALF was measured using Countess II (Thermo Fisher Scientific, San Diego, CA, USA). Additionally, to determine differential cell counts, the diluted pellets were attached to glass slides and then stained with Diff-Quik agent (Biozoa, Seoul, Republic of Korea). The numbers of eosinophils, lymphocytes, macrophages, and neutrophils were counted using a light microscope, and then the obtained ratios of differential cell counts were adjusted to the results of the total inflammatory cell counts.

### 2.8. Western Blot

To investigate the signaling pathway related to LTE treatment, the right lung was homogenized. Western blot was performed as previously described [14]. The primary antibodies were used as follows: p-p65 (dilution 1:1000, Cell signaling, Danvers, MA, USA), matrix-metalloproteinase 9 (MMP-9; dilution 1:1000, Cell signaling), cyclooxygenase2 (COX2; dilution 1:1000, Abcam, Cambridge, UK), hemooxygenase-1 (HO-1; dilution 1:1000, cell signaling), and β-actin (dilution 1:1000, cell signaling). The secondary antibody (Thermo Fisher Scientific) was diluted 1:3000 and used. The density of each protein band was measured using ChemiDoc (Bio-Rad, Hercules, CA, USA).

### 2.9. Histological Examination of Pulmonary Tissue

After sampling BALF, pulmonary tissues were fixed, and then a paraffin block was prepared. The paraffin block was sectioned (4 µm) and then stained with hematoxylin and eosin (Sigma-Aldrich, St. Louis, MO, USA). To explore the effects of LTE on the expression of HO-1, NF-κB, Nrf2, and 8-OHdG in ASD-exposed pulmonary tissue, immunohistochemistry was conducted using a commercial kit (Vector Laboratories, Burlingame, CA, USA) as previously described [15]. The following primary antibodies were used: anti-rabbit p-p65 (dilution 1:200, Thermo Fisher Scientific), anti-rabbit HO-1 (dilution 1:200, Abcam), anti-mouse Nrf2 (dilution 1:200, Santa Cruz, CA, USA), and anti-mouse 8-OHdG (dilution 1:200, Santa Cruz). Quantitative analysis of the pulmonary inflammation and density of protein band was conducted using IMT i-Solution (IMT i-Solution Inc., Vancouver, BC, Canada).

### 2.10. Measurement of Reactive Oxygen Species (ROS)

For ROS detection in BALF, a 2′, 7′-dichlorodihydrofluorescein (DCF) ROS/RNS assay kit (ab238535, Abcam) was performed according to the protocol of manufacturer. For intracellular ROS levels, NCI-H292 cells (CRL-1848, ATCC, Manassas, VA, USA) were incubated in a humidified chamber at 37 °C with 5% CO_2_ for 45 min with a 2′, 7′-dichlorodihydrofluorescein diacetate. Fluorescence was determined at 485 nm excitation/535 nm emission.

### 2.11. Immunofluorescence in NCI-H292 Cells

Immunofluorescence was conducted as previously described [10]. The primary antibodies were used as follows: NF-κB (cell signaling), MMP-9 (Abcam), and HO-1 (Abcam). The representative images were obtained using a confocal laser scanning microscope (ZEISS, Dresden, Germany).

### 2.12. Statistical Analysis

Data are expressed as the means ± standard deviation (SD). Statistical evaluation was conducted using analysis of variance (ANOVA) with Dunnett’s adjustment. The values of *p* < 0.05 and *p* < 0.01 were determined to be significant.

## 3. Results

### 3.1. Physiochemical Characterization of ASD

**Figure 1:**
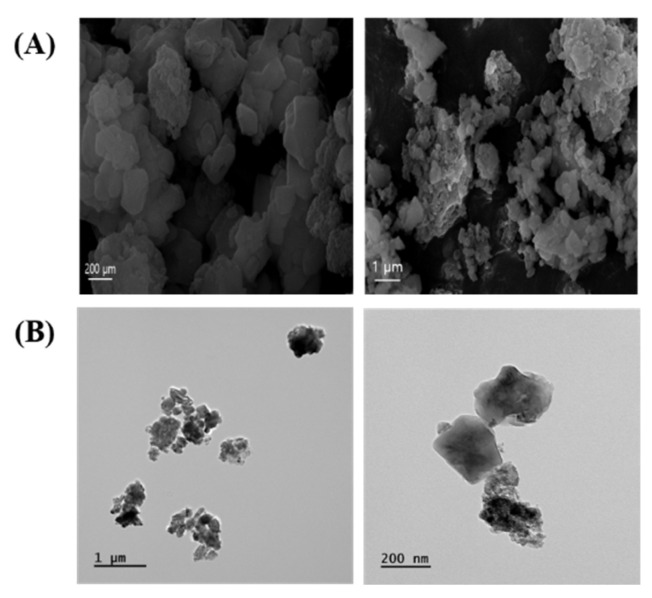
Morphology of ASD. (**A**) Morphology was measured by SEM. (**B**) Morphology was determined by TEM.

The morphology of ASD was mainly characterized by spherical shapes according to SEM (Figure 1A) and TEM (Figure 1B) analysis. ASD consisted of O 50.37%, Si 43.38%, Al 3.98%, Fe 1.88%, Ca 0.15%, Ti 0.13%, and Mg 0.11%.

### 3.2. Potential Target Gene Analysis

**Figure 2:**
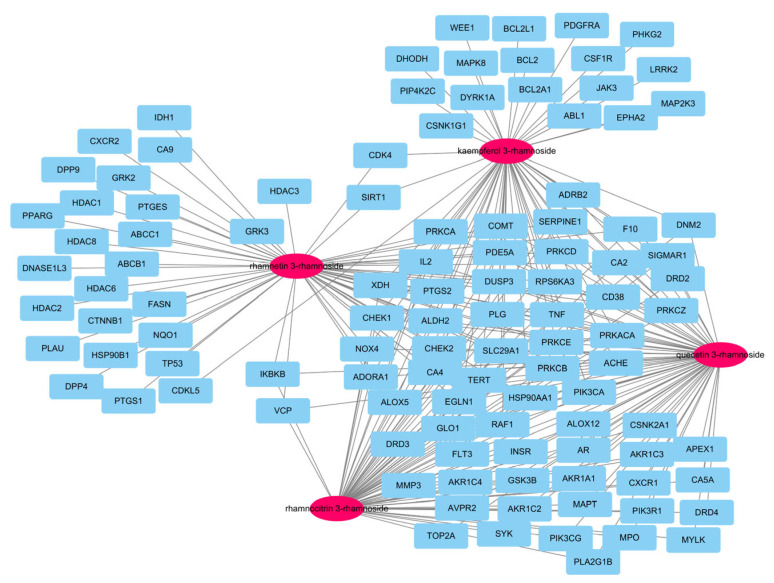
A network of 4 molecules (pink oval) and 109 potential target genes (blue rectangles). The network consisted of 113 nodes and 248 edges.

In our previous report, there were a total of 173 genes associated with the main small molecules, which are rhamnetin 3-rhamnoside, rhamnocitrin 3-rhamnoside, quercetin 3-rhamnoside, and kaempferol 3-rhamnoside [10]. A total of 109 potential target genes were identified among 6755 pneumonia-related genes retrieved from GeneCards DB (Supplementary Table S1), for a total of 173 genes (Supplementary Table S2). A total of 33 genes were associated with 4 main small molecules, which were ACHE, ADORA1, ALDH2, ALOX5, CA2, CA4, CD38, CHEK1, CHEK2, COMT, DUSP3, EGN1, F10, HSP90AA1, IL2, NOX4, PDE5A, PIK3CA, PLG, PRKACA, PRKCA, PRKCB, PRKCD, PRKCE, PRKCZ, PTGS2, RAF1, RPS6KA3, SERPINE1, SLC29A1, TERT, TNF, and XDH. The potential gene network of small molecules was constructed with 113 nodes and 248 edges (Figure 2).

### 3.3. Protein-Protein Interaction (PPI)

**Figure 3:**
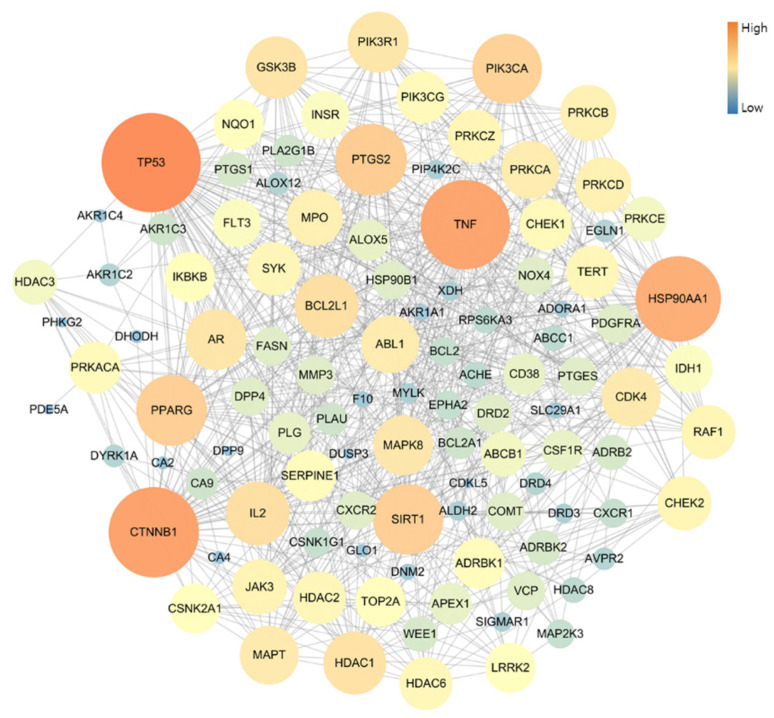
Topology analysis of PPI-associated pneumonia. The higher-degree genes are represented in large circles and orange color.

The PPI network of 109 potential pneumonia-linked genes was constructed with 107 nodes and 722 edges. For network topology analysis, the top 10 degree genes were TP53, CTNNB1, TNF, HSP90AA1, PTGS2, SIRT1, PPARG, PIK3CA, BCL2L1, and IL2; the top 10 betweenness centrality genes were TP53, CTNNB1, TNF, HSP90AA1, PTGS2, PPARG, PRKACA, MAPT, NQO1, and AR; and the top 10 closeness centrality genes were TP53, CTNNB1, TNF, HSP90AA1, PTGS2, PPARG, SIRT1, BCL2L1, PIK3CA, and GSK3B. As a result of analyzing three factors in the PPI topology, it was confirmed that a total of six genes, TP53, CTNNB1, TNF, HSP90AA1, PTGS2, and PPARG, were core genes in this PPI network (Figure 3).

### 3.4. Signaling Pathway Analysis

**Figure 4:**
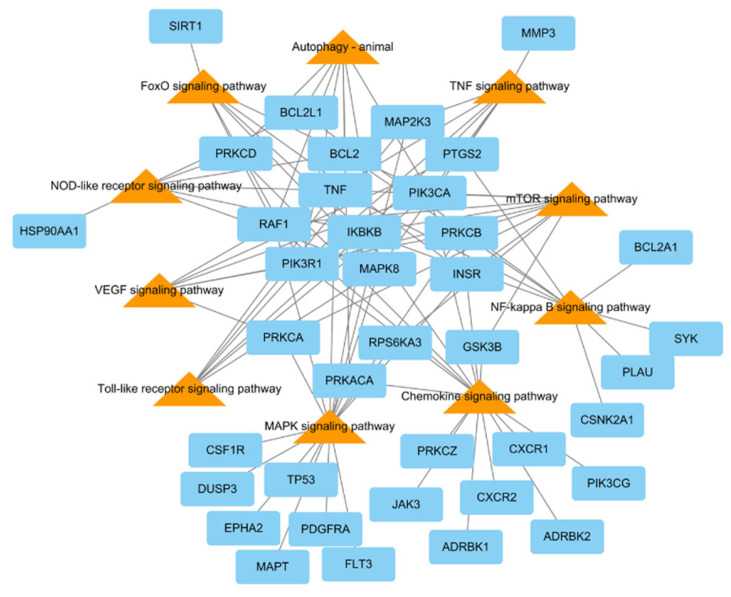
KEGG pathways related to pneumonia network consisting of potential target genes (blue rectangles) and KEGG pathways (orange triangles), with 48 nodes and 94 edges.

To research the signaling pathway related to the therapeutic effect of *L. tanakae* on pneumonia, we analyzed the KEGG pathway (*p* < 0.05). There were a total of ten pneumonia-related pathways, which were TNF signaling, MAPK signaling pathway, chemokine signaling, NF-κB signaling, mTOR signaling, VEGF signaling, autophagy-animal, Toll-like receptor signaling, FoxO signaling, and NOD-like receptor signaling. The genes related to more than five pathways were MAPK8, PRKCB, IKBKB, PIK3CA, PIK3R1, RAF1, and TNF, of which IKBKB was involved in eight pathways (Figure 4).

### 3.5. Effects of LTE on Inflammatory Cell Accumulation in Mice Exposed to ASD

**Figure 5:**
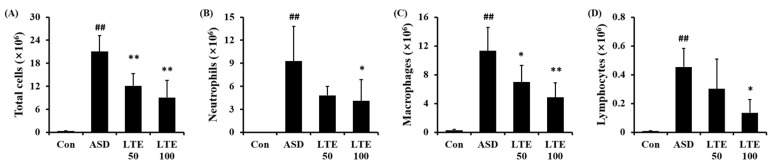
Effects of LTE on inflammatory cell accumulation in BALF from mice exposed to ASD. (**A**) Total cells, (**B**) neutrophil count, (**C**) macrophage count, (**D**) lymphocyte count. Con, PBS intranasal administration + PBS; ASD, ASD intranasal administration + PBS; LTE 50 and 100, ASD intranasal administration + LTE (50 and 100 mg/kg, respectively). The data are expressed as the mean ± SD (*n* = 5). ##, *p* < 0.01, vs. Con; *, ** *p* < 0.05 and <0.01, vs. ASD, respectively.

ASD exposure markedly elevated the total cell count in BALF in comparison to the control group (Figure 5A). By contrast, the LTE-administered groups showed obvious reductions in total cell count in BALF in comparison to the ASD group. The numbers of neutrophils, macrophages, and lymphocytes in BALF were remarkably elevated in the ASD group (Figure 5B,C,D, respectively). However, the LTE-administered groups showed declines in inflammatory cells in comparison to the ASD group, which was meaningfully observed in the LTE 100 group.

### 3.6. Effects of LTE on the Production of Inflammatory Cytokines and ROS in Mice Exposed to ASD

**Figure 6:**
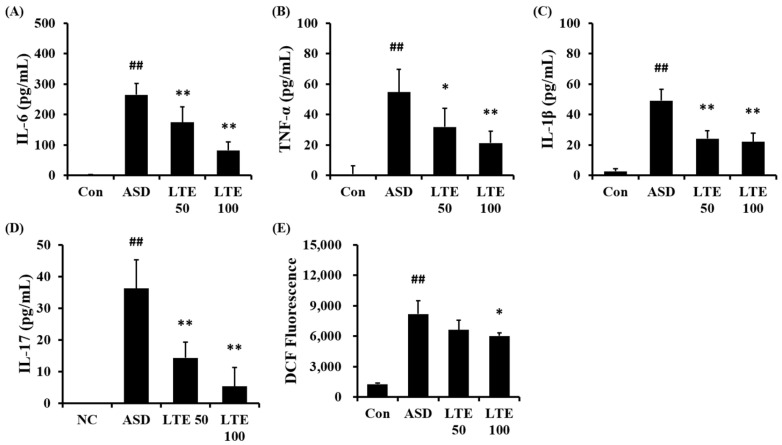
Effects of LTE on the production of inflammatory cytokines and ROS in mice exposed to ASD. (**A**) IL-6 level, (**B**) TNF-α level, (**C**) IL-1β level, (**D**) IL-17 level, (**E**) ROS production. Con, PBS intranasal administration + PBS; ASD, ASD intranasal administration + PBS; LTE 50 and 100, ASD intranasal administration + LTE (50 and 100 mg/kg, respectively). The data are expressed as the mean ± SD (*n* = 5). ##, *p* < 0.01, vs. Con; *, ** *p* < 0.05 and <0.01, vs. ASD, respectively.

ASD exposure obviously elevated the production of IL-6 in comparison to the control group (Figure 6A). But the LTE-administered groups exhibited meaningful reductions in the production of IL-6 in comparison to the ASD group, which was detected in a dose-dependent manner. In addition, the production of TNF-α, IL-1β, and -17 was obviously raised in the ASD group in comparison to the control group (Figure 6B,C,D, respectively). By contrast, the LTE-administered groups showed significant declines in TNF-α, IL-1β, and -17 in comparison to the ASD group, which was obviously detected. ASD exposure remarkably raised the production of ROS in comparison to the control group (Figure 6E). But the LTE100 group showed a decline in the production of ROS in comparison to the ASD group.

### 3.7. Effects of LTE on Inflammatory Process of Pulmonary Tissue from Mice Exposed to ASD

**Figure 7:**
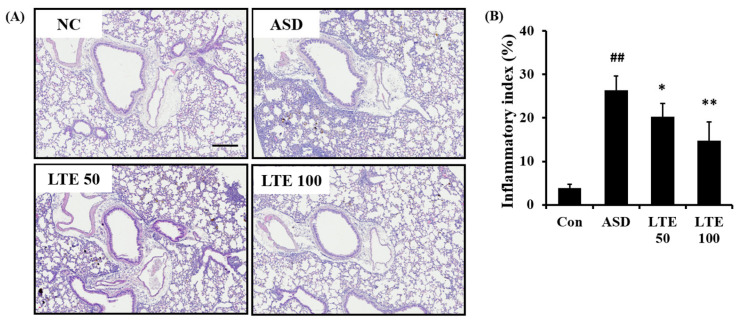
Effects of LTE on inflammatory responses of pulmonary tissue in mice exposed to ASD. (**A**) Representative figure for H&E-stained sections, (**B**) inflammatory index. Con, PBS intranasal administration + PBS; ASD, ASD intranasal administration + PBS; LTE 50 and 100, ASD intranasal administration + LTE (50 and 100 mg/kg, respectively). Scale bar indicates 100 μm. The data are expressed as the mean ± SD (*n* = 5). ##, *p* < 0.01, vs. Con; *, ** *p* < 0.05 and <0.01, vs. ASD, respectively.

ASD exposure caused extensive inflammatory responses in the pulmonary tissue in comparison to the control group (Figure 7A,B). However, the LTE-treated groups showed declines in the inflammatory process in pulmonary tissue compared with the ASD group, which was significantly observed in the LTE100 group.

### 3.8. Effects of LTE on Inflammatory Protein Expression in ASD Exposed Mice

**Figure 8:**
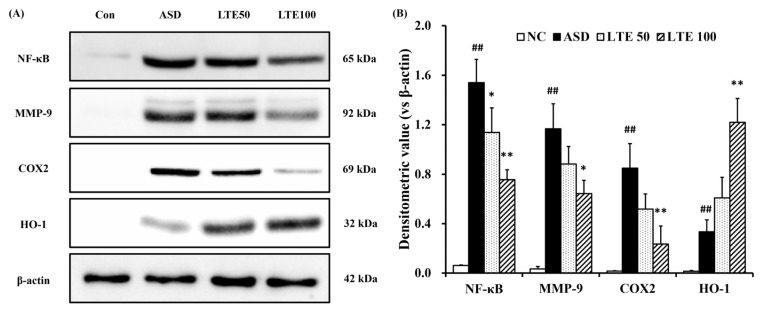
Effects of LTE on inflammatory protein expression in mice exposed to ASD. (**A**) Representative figure for protein expression, (**B**) densitometric value of protein expression. Con, PBS intranasal administration + PBS; ASD, ASD intranasal administration + PBS; LTE 50 and 100, ASD intranasal administration + LTE (50 and 100 mg/kg, respectively). The data are expressed as the mean ± SD (*n* = 5). ##, *p* < 0.01, vs. Con; *, ** *p* < 0.05 and <0.01, vs. ASD, respectively.

**Figure 9:**
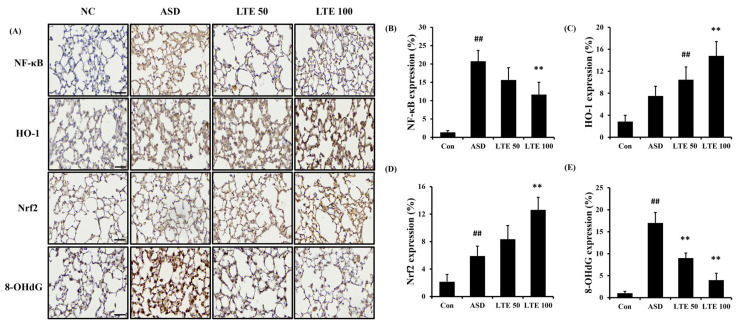
Effects of LTE on the expression of NF-κB, Nrf2, HO-1, and 8-OHdG in pulmonary tissue in mice exposed to ASD. (**A**) Representative figure for IHC, (**B**) NF-κB expression value, (**C**) Nrf2 expression value, (**D**) HO-1 expression value, (**E**) 8-OHdG expression value. Con, PBS intranasal administration + PBS; ASD, ASD intranasal administration + PBS; LTE 50 and 100, ASD intranasal administration + LTE (50 and 100 mg/kg, respectively). Scale bar indicates 50 μm. The data are expressed as the mean ± SD (*n* = 5). ##, *p* < 0.01, vs. Con; ** *p* < 0.01, vs. ASD, respectively.

ASD exposure induced the elevation of NF-κB expression in comparison to the control group (Figure 8A,B). By contrast, the LTE-administered groups showed decreased expression of NF-κB compared with the ASD group, which was obviously detected in the LTE100 group. The expression of MMP-9 and COX2 markedly increased with ASD exposure, which was reversed by LTE treatment. In addition, ASD exposure increased the HO-1 expression compared with the control group, and the LTE-administered groups had increased HO-1 expression compared to the ASD group. This was consistent with the results of ICH for pulmonary tissue. ASD exposure obviously increased the expression of NF-κB in pulmonary tissue in comparison to the control group (Figure 9A,B). By contrast, the LTE-administered groups showed meaningful declines in the expression of NF-κB in pulmonary tissue compared with the ASD group. In addition, the LTE-administered groups had elevated Nrf2 and HO-1 expression in pulmonary tissue compared with the ASD group, which was significantly observed in the LTE100 group (Figure 9A,C,D). The expression of 8-OHdG was elevated in the ASD group compared to the control group (Figure 9A). But the LTE-administered groups had remarkably decreased expression of 8-OHdG compared to the ASD group, which was obviously detected.

### 3.9. Effects of LTE on the ROS Production and Protein Expression in ASD-Stimulated NCI-H292 Cells

**Figure 10:**
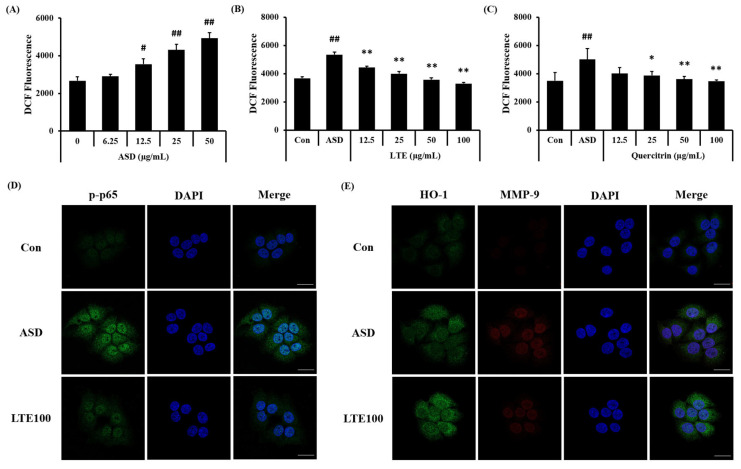
Effects of LTE and quercitrin on the ROS production and protein expression in ASD-stimulated NCI-H292 cells. ROS production in (**A**) ASD stimulated cells, (**B**) LTE-treated cells, and (**C**) quercitrin-treated cells. (**D**) Representative images for NF-κB, (**E**) representative images for HO-1 and MMP-9. Scale bar = 20 µm. The data are expressed as the mean ± SD (*n* = 3). #, ##, *p* < 0.05 and 0.01, vs. Con, respectively; *, ** *p* < 0.05 and 0.01, vs. ASD, respectively.

ASD exposure induced ROS generation in NCI-H292 cells, which was detected in a concentration-dependent manner (Figure 10A). Treatment of LTE and quercitrin effectively decreased the production of ROS induced by ASD exposure (Figure 10B,C). In addition, its translocation into the nuclei of ASD-stimulated cells was increased compared to that of non-stimulated cells (Figure 10D). By contrast, the LTE treatment groups had significantly decreased translocation of NF-κB compared to ASD stimulated cells. Exposure to ASD induced the increased expression of HO-1 and MMP-9 compared to non-stimulated cells (Figure 10E). The expression of HO-1 was markedly increased compared to the ASD-stimulated cells. However, the expression of MMP-9 was decreased compared to the ASD-stimulated cells.

## 4. Discussion

As industrialization accelerates, air pollution is emerging as a social problem. In particular, ASD, generated from Northeast Asia, contains various industrial substances, heavy metals, nanoparticles, and chemicals. It affects China, Korea, and Japan, and causes various respiratory diseases [3]. Here, we assessed the sanative effects of LTE in an ASD-exposed airway inflammation model and investigated its mechanism of action via network pharmacological analysis and protein assay. First, we analyzed four ingredients of LTE, including afzelin, quercitrin, rhamnocitrin 3-rhamnoside, and rhamnetin 3-rhamnoside. In an in vivo experiment, LTE treatment caused a significant decline in the inflammatory cell count and inflammatory cytokines in BALF from mice exposed to ASD, along with decreases in inflammatory cell accumulation in pulmonary tissue. Furthermore, LTE treatment markedly decreased the protein expression, including NF-κB, MMP-9, and COX2, related to the inflammatory process and elevated the HO-1 expression, acting as an antioxidant protein. This corresponded to the data of the network pharmacological analysis.

Because ASD consists of not only sand dust, but also various toxic stimuli, when exposed to the respiratory tract, it induces an extensive inflammatory response in the lung tissue [2]. In previous studies, ASD exposure has produced a significant amount of inflammatory cytokines, which induced inflammatory cell accumulation in pulmonary tissue and aggravated underlying respiratory diseases [6]. In this study, ASD exposure caused the elevation of inflammatory cytokines in BALF. But LTE treatment meaningfully decreased the production of inflammatory cytokines caused by ASD exposure, which was accompanied by declines in inflammatory cell count in BALF and airway inflammation in pulmonary tissue. Considering these results, LTE suppressed the pulmonary inflammation induced by ASD exposure.

Airway inflammation is a complex process involved in several signaling transduction. Particularly, NF-κB is an important signaling pathway in inflammatory process [16]. It is activated by several stimuli, such as lipopolysaccharides, cytokines, and chemokines, which induce its phosphorylation with the activation of IκB [17]. Then, translocation into the nucleus proceeds with the transcription of inflammatory associated genes, resulting in inflammatory cell infiltration into damaged lesions [18]. ASD exposure also acts as stimulus to activate NF-κB, which induces airway inflammatory responses [19]. Therefore, the suppression of NF-κB is considered as a crucial strategy to control ASD-induced airway inflammation. LTE administration markedly inhibited the expression of NF-κB induced by ASD exposure, which was accompanied with a decline in the expression of COX2 and MMP-9. This indicates that the curative properties of LTE against ASD-related pulmonary inflammation are linked with the decrease in the expression of NF-κB. Additionally, ASD exposure induced oxidative stress, which was involved in the alteration of harmful responses in the respiratory tract [6]. ASD exposure caused an imbalance of oxidants/antioxidants via the excessive production of ROS [20]. These events eventually induced a reduction in the antioxidant system, resulting in aggravated inflammation and apoptosis [21]. Therefore, several antioxidant systems exist to protect against oxidative stress. Of the antioxidant systems, HO-1 is an important molecule to reduce oxidative stress [22]. HO-1 is expressed via several irritations that cause ROS production and then diminish oxidative stress by catalyzing the degradation of heme to biliverdin/bilirubin, which act as antioxidants [23]. In a previous study, the overexpression of HO-1 induced a reduction in oxidative stress, but a deficiency of HO-1 caused an elevation of oxidative stress via excessive ROS production [24]. In the present study, LTE treatment markedly increased HO-1 expression compared with the ASD group. This indicates that LTE treatment effectively attenuated oxidative stress caused by ASD exposure.

LTE is a traditional herbal remedy in China, Japan, and Korea to control several inflammatory disorders. We detected four ingredients, including afzelin, quercitrin, rhamnocitrin 3-rhamnoside, and rhamnetin 3-rhamnoside, in LTE using an HPLC system. Four ingredients were associated with the therapeutic effects of LTE due to their pharmacological properties. Afzelin has antioxidative and anti-inflammatory properties and exerts suppressive effects against particulate matter-induced inflammatory skin diseases and ultraviolet radiation-induced damage [25,26]. Quercitrin has shown antioxidative and anti-inflammatory properties in various experiments, and is linked with the suppression of NF-κB and overexpression of HO-1 [27]. Rhamnocitrin 3-rhamnoside and rhamnetin 3-rhamnoside showed strong antioxidant properties in our previous study [10]. Thus, our experiment is strongly supported by these previous studies.

In this study, PBS was used as a vehicle control. However, the solubility, stability, and bioavailability of LTE were not evaluated before using PBS as a vehicle control. Since these matters may affect the efficacy of the test material, it is considered essential to perform animal experiment. If somewhat negative results occur regarding the pharmacological properties of test material, another vehicle control should be considered. These matters will need to be sufficiently reflected in further experiments.

Based on the data of network pharmacology analysis, the top 10 pathways (according to *p*-value) related to pneumonia were as follows: chemokine signaling, MAPK signaling, TNF signaling, FoxO signaling, Toll-like receptor signaling, autophagy-animal, VEGF signaling, NF-κB signaling, mTOR signaling, and NOD-like receptor. Particularly, TNF and NF-κB signaling may be linked in inflammatory processes. The signaling of TNF and NF-κB stimulated by various irritation could lead to an inflammatory process via the production of inflammatory cytokines [28]. Additionally, TNF, FoxO, and NF-κB were involved in pathological conditions associated with ROS generation. The signaling of TNF and NF-κB could cause the progression of pulmonary inflammation due to excessive ROS generation [29]. On the other hand, FoxO signaling could activate antioxidant defense mechanisms such as HO-1, Nrf2, and superoxide dismutase to scavenge ROS [30]. Therefore, the curative properties of LTE against ASD-caused pulmonary inflammation may be linked with the elevation of HO-1 expression and inhibition of NF-κB expression.

## 5. Conclusions

Overall, LTE treatment effectively attenuated pulmonary inflammatory process induced by ASD exposure, which was related to the elevation of HO-1 expression and inhibition of NF-κB expression. Thus, LTE may be used as a potential therapeutic remedy for treating pulmonary inflammation.

## Data Availability

Data are contained within the article or Supplementary Material.

## Supplementary Materials

The following supporting information can be downloaded at: https://www.mdpi.com/article/10.3390/antiox13040419/s1, Table S1: Pneumonia-related genes in Gene Cards database; Table S2: Information of small molecules and genes in *Loranthus tanakae*.
